# Effective Immobilization of Novel Antimicrobial Peptides via Conjugation onto Activated Silicon Catheter Surfaces

**DOI:** 10.3390/pharmaceutics16081045

**Published:** 2024-08-06

**Authors:** Irem Soyhan, Tuba Polat, Erkan Mozioglu, Tugba Arzu Ozal Ildenız, Merve Acikel Elmas, Sinan Cebeci, Nihan Unubol, Ozgul Gok

**Affiliations:** 1 Department of Medical Biotechnology, Acibadem Mehmet Ali Aydinlar University, Atasehir, 34752 Istanbul, Turkey; iremsoyhan@gmail.com (I.S.); erkan.mozioglu@acibadem.edu.tr (E.M.); sinan.cebeci@acibadem.edu.tr (S.C.); 2 Department of Medical Microbiology, Acibadem Mehmet Ali Aydinlar University, Atasehir, 34752 Istanbul, Turkey; tuubapolat24@gmail.com; 3 Department of Biomedical Engineering, Acibadem Mehmet Ali Aydinlar University, Atasehir, 34752 Istanbul, Turkey; tugba.ildeniz@acibadem.edu.tr; 4 Department of Histology and Embriology, School of Medicine, Acibadem Mehmet Ali Aydinlar University, Atasehir, 34752 Istanbul, Turkey; merve.elmas@acibadem.edu.tr; 5 Medical Laboratory Technician Program, Vocational School of Health Services, Acıbadem Mehmet Ali Aydınlar University, Atasehir, 34752 Istanbul, Turkey

**Keywords:** surface modification, antimicrobial peptides, conjugation, thiol-ene chemistry, peptide synthesis

## Abstract

Antibiotic-resistant microorganisms have become a serious threat to public health, resulting in hospital infections, the majority of which are caused by commonly used urinary tract catheters. Strategies for preventing bacterial adhesion to the catheters’ surfaces have been potentially shown as effective methods, such as coating thesurface with antimicrobial biomolecules. Here, novel antimicrobial peptides (AMPs) were designed as potential biomolecules to prevent antibiotic-resistant bacteria from binding to catheter surfaces. Thiolated AMPs were synthesized using solid-phase peptide synthesis (SPPS), and prep-HPLC was used to obtain AMPs with purity greater than 90%. On the other side, the silicone catheter surface was activated by UV/ozone treatment, followed by functionalization with allyl moieties for conjugation to the free thiol group of cystein in AMPs using thiol-ene click chemistry. Peptide-immobilized surfaces were found to become more resistant to bacterial adhesion while remaining biocompatible with mammalian cells. The presence and site of conjugation of peptide molecules were investigated by immobilizing them to catheter surfaces from both ends (C-Pep and Pep-C). It was clearly demonstrated that AMPs conjugated to the surface via theirN terminus have a higher antimicrobial activity. This strategy stands out for its effective conjugation of AMPs to silicone-based implant surfaces for the elimination of bacterial infections.

## 1. Introduction

Antimicrobial resistance (AMR) is a severe problem that compromises the effectiveness of medications against bacterial infections. AMR can be caused by antibiotic misuse, overuse of antimicrobial drugs in agriculture and the animal sector, and mutations in microorganisms [1]. Because AMR reduces the efficacy of used antimicrobial drugs, it raises the risk of persistent infection and its spread. To overcome AMR, antimicrobial peptides (AMPs) have been designed to provide promising results that are less prone to bacteria gaining resistance because of the fact that their pharmacodynamic properties and working processes usually differ from those of common antibiotics [2].

While antibacterial drugs are classified into two groups based on their type of action, bacteriostatic orbactericidal, they appear to differ in their chemical structures, activity spectrum, mode of action, and level of resistance to antibiotics [3]. Polymers with quaternary nitrogen groups, poly-L-lysine, and halamines in their chemical structure have the potential to kill or inhibit the growth of microorganisms [4], but their high molecular weight and limited water solubility may pose challenges for large-scale medical applications [5]. As an innovative alternative, synthetic antimicrobial peptides (AMPs) with α-helices and/or β-sheets in their secondary structures have emerged as crucial defensive agents of the innate immune system against pathogens [6]. In general, they are less than 100 amino acids long and have a net positive charge due to the presence of cationic lysine (Lys, K) and arginine (Arg, R) amino acid residues [7]. The cationic and hydrophobic amino acid residues give the AMPs sufficient amphipathicity to interact with the anionic bacterial membrane [8]. Based on this electrostatic interaction, AMPs have been shown to be absorbed into the bacterial membrane and then permeate inside, disrupting their integrity [8,9].

AMPs with less toxicity have great potential to provide superior antimicrobial features compared to the present conventional antibiotics. Shown as more effective in in vitro and in vivo studies, AMPs emerge as powerful medications to dispose of antibiotic-resistant bacteria, with a non-reversible inhibition process [9,10,11]. The most general classification for the production strategy of AMPs can be divided into three categories: in vivo production, cell-free systems, and chemical synthesis. The first category has limitations during the extraction and isolation processes due to the toxicity of host proteases [12,13]. In cell-free protein production systems, the protein synthesis machinery obtained from crude cell lysate is used [14], which prevents the incorporation of non-natural or chemically modified amino acids [15]. However, solid-phase peptide synthesis (SPPS) has grown in popularity in recent years as a method for preparing chemically modified peptides by allowing synthetic and D-form aminoacids to be attached in a predetermined sequence with high purity. Because no host cell or natural protein synthesis machinery is used during SPPS, the unnatural amino acid can be easily incorporated [16]. The basic idea is to elongate the peptide chain from a solid support, such as a resin, using a series of deprotection and coupling cycles for each additional amino acid. After reaching the desired length in the direction from the C to the N terminal, the peptide is cleaved from the solid support, and all excess reagents and byproducts are removed [16].

Particularly for reducing the incidence of health care-associated infections or biomaterial-associated infections, synthetically prepared and chemically modified AMPs seem to enable removing the biofilm produced by the antibiotic-resistant bacteria on implantable medical devices. Unlike traditional antibiotic-based treatments, some AMPs are shown to preserve better stability under physiological conditions and are thus more effective against aggressive infections [17]. Catheter-associated urinary tract infections (CAUTIs) appear to be common due to the frequent catheterization periods, which range from a few days to weeks or even permanent implantation [18]. The most common CAUTI-causing agents include *Escherichia coli* (*E. coli*), *Staphylococcusaureus* (*S. aureus*), and *Pseudomonas aeruginosa* (*P. aeruginosa*), etc. [19]. The main strategy to eliminate the risk of infection originating from these catheters is either their preparation from antimicrobial materials, which results in large synthesis research with a limited source, or surface modifications to make them interact less with the patient’s own floral microorganisms orexogenous sources like contaminations, so that it would be possible to remove the possibility of bacterial adhesion, which would probably turn into a biofilm, especially on hydrophobic surfaces like silicone [20].

Surface modification strategies have been widely used to change the physical, chemical, and morphological properties of catheters to effectively prevent and eliminate CAUTIs. While coating the surface with hydrophilic polymers such as poly ethylene glycol [21] or antibiotics such as penicillin and ampicillin [22] reduces protein adsorption, no permanent solution is achieved due to the removal of the coating layer and the development of bacterial resistance [23,24]. However, immobilization of antimicrobial molecules such as AMPs has grown in popularity as a more effective method of preventing biofilm formation [25,26]. Using functional moieties that can be incorporated into the chemical structure of AMPs such as the addition of amine, thiol, maleimide, or NHS-activated carboxylic acid groups, they can be conjugated to catheter surfaces [27,28]. In particular, silicone-based catheter surfaces can be introduced with hydroxyl groups upon chemical treatments such as UV or ozone treatment, allowing for modifications with silane group-containing molecules [29].

The literature reveals the attachment of various antimicrobial molecules on silicone catheter surfaces using very well-known reactions such as amidation, click reactions, and nucleophilic 1,4 Michale addition reactions. In this study, novel antibiotic-resistant antimicrobial peptides with a sequence of ‘RLLLRLLRRLLRLLLR’ were prepared via the SPPS method and named as ‘PEP’ forshort. They have been synthesized with an additional cystein aminoacid at either end (C-PEP or PEP-C), so that they could be immobilized on silicone catheter surfaces from either end using thiol-ene click chemistry and evaluated for their active end [30]. For the conjugation reaction, the catheter surface was activated using UV/ozone treatment and then modified with an allyl functional group containing a silane molecule as a linker. AMPs were attached to these allyl groups using a UV light-mediated radical addition reaction with DMPA as a photoinitiator [31]. In addition to extensive characterization of the peptide-conjugated surface, the cytotoxicity toward mammalian cell lines and antimicrobial activity against commonly seen bacteria strains were investigated. The obtained data indicated that AMPs were successfully immobilized on the catheter surface, preserving a high potential for use as a bioactive medical tool in eliminating hospital-associated infections.

## 2. Materials and Methods

### 2.1. Thiolated Antimicrobial Peptides

*Solid Phase Peptide Synthesis (SPPS) of C-AMPs.* The peptides (C-PEP and PEP-C) were synthesized with The Liberty Blue^TM^ Automated Microwave Peptide Synthesizer (CEM) [32]. Rink Amide Protide was used as the resin and the scale of synthesis was 0.143 mmole, swollen in dimethylformamide (DMF) for at least 30 min prior to peptide synthesis. The amino acid conformationsfor the antimicrobial peptides were Arginine (Arg, R) and Cysteine (Cys, C) in L form, Leucine (Leu, L) in D form. A total of 4.68 g of R in L form was weighed and dissolved in 36 mL of DMF. Similarly, 2.2 g of L in D form and 0.36 g of C in L form were weighed and dissolved in DMF. N,N′-Diisopropylcarbodiimide (DIC) was used as activator and 2.2 mL of DIC was completed to 28 mL with DMF. For an activator base, oxyma was used, and 1.0 M oxyma was prepared in 14 mL of DMF. Deprotection cocktail was prepared with 20% Piperdine (*v*/*v*) in DMF. At the end of the synthesis process, the obtained peptide was cleaved from the resin with the Razor^TM^ Peptide Cleavage System (CEM). The cleavage cocktail consists of 4.75 mL of trifluoroacetic acid (TFA), 125 µL of triisopropylsilane (TIS), and 125 µL of distilled water (ddH_2_O). The peptide cleavage was carried out at 38 °C for 45 min. The cleaved peptide was precipitated in cold diethyl ether and then centrifuged at 4000 rpm for 3 min. After carefully discarding the ether phase, the peptide pellet was left to dry.

*Quantification and Purification of C-AMPs.* Peptides were purified by using the 1260 Infinity Quaternary LC (Agilent Technologies, Santa Clara, CA, USA) systems with prep RP-HPLC hydrophobic 6 µm C18 column (Agilent VariTide RPC 250 × 10 mm ID). Purified C-AMPs were characterized with the 6420 Triple Quad LC/MS (Agilent Technologies) system with mobile phase of ACN with 0.05% *v*/*v* formic acid (FA). Samples were directly injected into a mass detector in scan mode between 50 and 2200 mass (m) ranges for 1 min, single ion monitoring (SIM) mode for 3 distinct charge (z) ions.

*Minimal Inhibition Concentration (MIC) Assay. E. coli NTCC13846*, *E. coli ATCC25922*, *S. aureus ATCC 29213*, *S. aureus ATCC25923*, and *MRSA*, were incubated in Mueller–Hilton Broth (MHB) overnight at 37 °C [33]. The OD600 measurement was conducted with the Gen5 Micro plate reader, on the overnight bacteria incubations which were 0.5 McFarland (1.0 × 10^8^ cfu/mL) adjusted to 0.1. OD600-adjusted bacteria cultures were diluted to a 1:100 ratio with MHB. Serial dilutions of C-AMPs were prepared in MHB in 96-well plates and 5 µL of bacterial suspension was added into each well that contained different dilutions of C-AMPs. The highest concentration of the AMPs was 128 µg/mL. As control groups, ampicillin and the non-thiolated version of the C-AMPs were used as positive controls. All bacteria plates were incubated for 18–20 h at 37 °C and all the C-AMPs treatments were conducted in triplicate.

*Hemolytic Assay*. The hemolytic activity levels of prepared AMPs were determined by an assay with slight modifications from the literature [34]. Sterile Tris-Saline (10 mM Tris, 150 mM NaCl pH 7.2) solution was prepared for the hemolytic assay. A total of 30 µL of fresh blood and 10 mL of Tris-Saline solution were mixed well together and were then centrifuged at 1500 rpm for 5 min. The supernatant was discarded, and the pellet was dissolved with 10 mL of Tris-Saline solution; then, the centrifugation step was repeated. The red blood cell (RBC) pellet was dissolved with 10 mL of Tris-Saline solution and 100 µL was added into each well of a 96-well plate. On top of the RBCs, 100 µL of serial diluted C-AMPs were added. As a complete lysis control, Triton X-100 was used. The plate was incubated at 37 °C for 30 min. After incubation, the plate was centrifuged at 1500 rpm for 10 min. The supernatants were transferred into a new 96-well plate and measured at 414 nm.

In vitro *Cytotoxicity Assay*. Mouse fibroblast 3T3 (ATCC CRL-1658), human epidermal keratinocyte HaCat (ATCC PCS-200-011), and human cervix epithelial adenocarcinoma HeLa (ATCC CCL-2) cell lines were incubated with Dulbecco’s modified Eagle media (DMEM). For MTT cytotoxity assay (Roche), 5 × 10^4^ cells with a volume of 100 µL per well were seeded in a 96-well plate. Serial dilutions of C-AMPs 64–0.25 µg/mL were applied to the cells. Magainin II (Mag2), a known antimicrobial peptide that has a non-toxic nature and the non-thiolated version of the C-AMPs which is PEP, were used at the same concentrations as a positive control of the C-AMPs. C-AMPs-applied plates were incubated for 18 to 24 h at 37 °C, 5% CO_2_. After incubation, to measure the toxic effect of the C-AMPs, the MTT protocol was applied. The plates were incubated (37 °C, 5% CO_2_) overnight. After overnight incubation, the purple formazan crystals were solubilized, and the absorbance measurement of the samples was performed at 550 nm and 690 nm with Thermo Scientific Varioskan Flash plate reader [35].

### 2.2. Surface Modification of Silicone Catheter

*Hydroxylation of Silicone Catheter Surface By UV/Ozone Treatment*. The medical silicone catheter (Rüsch Brillant) was cut into approximately 0.5 mm pieces and cleaned with equal volumes of hydrogen peroxide (H_2_O_2_) and sulfuric acid (H_2_SO_4_) of piranha solution for 20 min and washed with distilled water. Catheter pieces were later dried for 1 h at 70 °C under vacuum [29]. Hydroxylation of catheter surfaces was performed using the ProCleaner^TM^ unit (Bioforce Nanosciences, Ames, IA, USA). The sample holder part inside was placed at a 0.3125 inch distance from the pedestal to the UV lamp. Later, the system was closed and the UV lamp was turned on at anintensity of 19.39 mW/cm^2^. The exposure of UV/ozone to the samples was set for 2 continuous hours. After the removal of the sample from the instrument, it was dipped into 2 mM reduced L-Glutathione (GSH) solution prepared in ddH_2_O for 30 min at 25 °C in order to get rid off the remaining radicals due to UV/ozone treatment [36].

*Attachment of the Linker ‘ATS’*. Surface-activated silicone catheter pieces were treated with Allytrimethoxysilane solution (5% ATS, 5% dH_2_O, and 90% ACN) for 4 h on a magnetic stirrer at room temperature [37]. After 4 h of intermediate molecule ATS treatment, silicone catheter pieces were removed and washed with 100% ACN and acetone, respectively. The washed silicone catheter pieces were dried in air for further C-AMPs immobilization.

*Free-Radical Conjugation Method of C-AMPs to the Modified Catheter Surface*. Then, 1 mg/mL C-AMPs were prepared in 50% ACN with DMPA which corresponds to the 0.1 molar equivalent of C-AMP. ATS-bounded catheter surfaces were placed into the C-AMPs immobilization solution carefully without damaging the modified outer surface of the silicone catheters and mixed under a UV lamp (365 nm) for 1 h with a magnetic stirrer [38]. Afterwards, the peptide-bounded catheter pieces were removed, washed with distilled water, and dried.

*Surface Characterization*. *Chemical Characterization*: The surface chemistry of the C-AMPs-immobilized catheter surface was measured with the Thermo^TM^ Nicolet iS10 FT-IR (Thermo Fisher, Waltham, MA, USA) to determine the presence of the amide bonds [29]. The C-AMPs-immobilized silicone catheter surfaces were placed in contact with the attenuated total reflection (ATR) sampling accessory as the modified face. Each sample spectrum was recorded from 400 to 4000 cm^−1^ with 32 scans at room temperature. *Physical Characterization:* The wettability of the modified catheter surfaces was analyzed by contact angle measurement technique. The static water contact angle (CA°) and the surface hydrophobicity measurements of the C-AMPs-immobilized and unbounded silicone PDMS surfaces were performed with Attention Theta Lite Contact Angle (Biolin Scientific, Gothenburg, Sweden). In total, 0.5 µL of water was dropped on the modified and unmodified silicone PDMS surfaces [39]. The images of the dropped water droplet were taken with a high magnification camera and the measurements were analyzed with the OneAttension software. *Morphological Characterization*: The semi-quantitative elemental analysis was performed with scanning electron microscopy (SEM) (Thermo Quattro S, Waltham, MA, USA, MAPS 3 Software) coupled with energy dispersive X-ray spectroscopy (EDS) (EDAX) by determining the elemental ratios of carbon (C), oxygen (O), nitrogen (N), and sulfur (S) atoms [40].

### 2.3. Antimicrobial Activity Test of C-AMP-Immobilized Silicone Catheter Surfaces

*Colony Counting*. *E. coli ATCC 25922* and *S. auresus ATCC 25923* were cultivated overnight in MHB at 37 °C. OD600 of the bacteria was adjusted to 0.1 which is equivalent to 1.0 × 10^8^ cfu. Bacteria inoculum was diluted to 1.0 × 10^5^ cfu/mL with MHB. C-AMP-immobilized catheter pieces were placed in 40 µL of a bacteria suspension as the coated surface would be in contact with it. The uncoated silicone catheter was used as a control. The inoculum-contacted, C-AMP-coated, and uncoated catheters were incubated 40 min at 37 °C. Afterwards, 10 µL of the inoculum was withdrawn and 8 serial dilutions were performed in the ratio of 1:10 with MHB. In total, 10 µL of serially diluted inoculum samples were dropped on MH agar and incubated overnight at 37 °C for the cfu determination [39]. To discover the effect of C-Pep-immobilized catheter surface on *E. coli ATCC 25922*, 1.0 × 10^3^ cfu of the *E. coli* bacterial strain was dropped onto C-Pep-immobilized silicone catheter surfaces and incubated at 37 °C. After incubation, glutaraldehyde solution (50% in water) was dripped onto the silicone catheter pieces with bacteria on them and left to fix for 1 day. The fixed surfaces are coated with gold (Au/Pb) and analyzed with scanning electron microscopy (SEM) under a low vacuum, 20,000 kV and 50 Pa.

## 3. Results

### 3.1. Synthesis of Thiolated Antimicrobial Peptides (C-AMPs)

**Figure 1:**
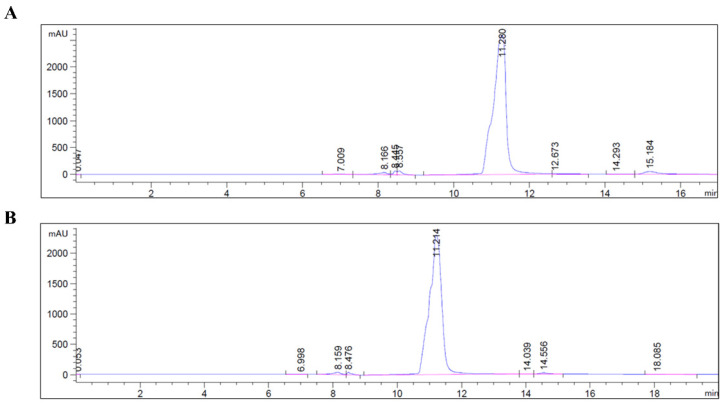
HPLC chromatograms for thiolated AMPs. (**A**) C-Pep purity as 97%; (**B**) Pep-C purity as 94%.

*Purification and characterization.* TheC-AMPs (peptides) were initially run with a general gradient for 30 min to scan for impurities and understand the polarity, which was detected around 60–70%. For C-PEP, the purity was calculated as 97% (Figure 1A), and for PEP-C the purity was 94% (Figure 1B).

**Figure 2:**
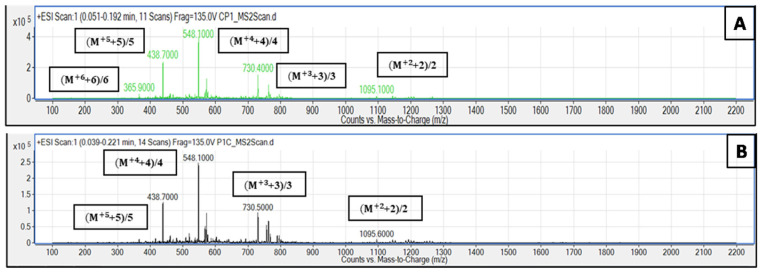
LC-MS/MS results in positive scan mode. (**A**) C-PEP, (**B**) PEP-C.

The samples were run in both negative and positive scan mode, in which the fragments were clearly seen in positive scan mode (Figure 2). For the *m*/*z* values of the C-Pep antimicrobial peptide in the positive scan mode, the five peaks correspond to +2, +3, +4, +5 and +6 charged fragments. For Pep-C, four fragments were detected in positive scan mode, and they correspond to the +2, +3, +4 and +5 charged fragments.

*Minimal Inhibitory Concentration (MIC).* The MIC values for both thiolated and non-thiolated versions of PEP are given in the table below (Table 1). When comparing the MIC results, non-thiolated PEP has better efficacy against the thiolated versions (C-PEP and PEP-C), which might be due to the fact that the free thiol coming from Cystein aminoacid might lead to a decrease in the stability. However, in this study, C-PEP and PEP-C were designed for their attachment to the catheter surface, so the MIC comparison between them is more significant.

C-PEP had a MIC value of 2 µg/mL against *E. coli ATCC 25922*, while PEP-C had 2 µg/mL. C-PEP was found to be more efficient than PEP-C and ampicillin, which hadan MIC value of 8 µg/mL. For all bacteria strains used in MIC, the value of PEP-C was found to be the same, which was 8 µg/mL. The MIC value of C-PEP was found to change between 1 µg/mL and 2 µg/mL. This means that C-PEP is four times more effective than PEP-C against *E. coli ATCC 25922, S. aureus ATCC 25923*, and *MRSA*; and eight times more effective against *E. coli NCTC 13846* and *S. aureus ATCC 29213*.

**Figure 3:**
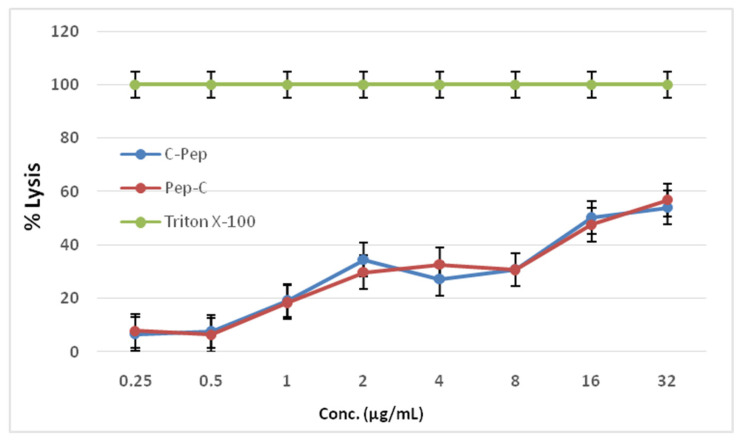
Lysis results of synthesized peptides on fresh human red blood cells.

*Hemolytic Activity*. The hemolytic activity of the thiolated peptides were measured with freshly collected human red blood cells. For 100% lysis, Triton-X-100 was used; the positive control and hemolysis values of 0.25–32 µg/mL concentrations of C-AMPs are given in the figure below (Figure 3). From the results, C-AMPs reached a HC50 value of around 16 µg/mL, which means the MIC values of both C-AMPs are lower than the HC50 values.

**Figure 4:**
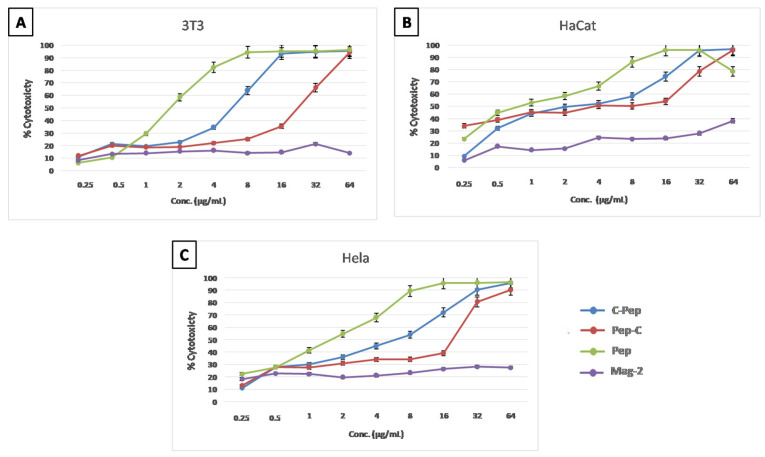
Cytotoxicity results. (**A**) Mouse fibroblast cell line 3T3; (**B**) human keratinocyte cell line, HaCat; (**C**) human cervical adenocarcinoma, HeLa.

*Cytotoxicity (MTT) results of C-AMPs*. The toxicity effects of the C-AMPs were analyzed in three different cell types, and for controls the non-thiolated PEP and Magainin-2, a known non-toxic AMP, were used. The cytotoxicity levels of PEP-C for the mouse fibroblast cell line 3T3 (Figure 4A) was less toxic at a higher concentration compared to C-PEP, PEP and Mag-2. Until 1 µg/mL, the toxicities of PEP-C and C-PEP are the same, but above that, C-PEP becomes slightly more toxic than PEP-C. C-PEP reaches the IC50 value after 4 µg/mL and PEP-C passes IC50 after the concentration of 16 µg/mL.

For the immortal human keratinocyte cell line (HaCat) at lower concentrations until 1 µg/mL, C-PEP was found to be less toxic than PEP-C (Figure 4B). The toxicity of PEP-C slowly increased as the peptide concentration increases until 16 µg/mL; after 16 µg/mL, the toxicity increased rapidly. At the concentration of 0.25 µg/mL, between non-thiolated PEP, C-PEP, and PEP-C, C-PEP was found to be the least toxic. The toxicity profiles of the non-thiolated PEP and C-AMPs were analyzed in the HeLa cell line. The toxicity of PEP-C was lower than 50% up to 16 µg/mL. At 32 µg/mL, the toxicity level of PEP-C increases to 80%. The toxicity of C-PEP increases slowly as the peptide concentration increases. C-PEP reaches 50% toxicity level after 4 µg/mL (Figure 4C).

### 3.2. Characterization of C-AMP-Conjugated Catheter Surfaces

*Chemical characterization: FT-IR Spectroscopy.* ATR/FT-IR spectrometry measurement was used to confirm the immobilization of C-AMPs on the medical silicone catheter surfaces. The ATR/FT-IR measurement was performed with 32 scans between 400 to 4000 cm^−1^. The FT-IR spectrum of the untreated silicone catheter (Figure S3) shows the characteristic peaks belonging to silicone, and a strong peak around 1074 cm^−1^ belonging to the stretching vibration of the Si-O-Si bond. There are two peaks belonging to Si-CH_3_, one is around 1400 cm^−1^ due to the symmetric stretching caused by CH_3,_ and the other one is at 1258 cm^−1^, belonging to the asymmetrical stretching of CH_3_. Around 2900 cm^−1^ there is a peak that belongs to the C-H stretching. After the UV/O_3_ treatment and incubation in the 2 mM GSH solution, a broad peak seems to appear at around 3500–3000 cm^−1^ that belongs to the hydroxyl groups (OH) (Figure S4).

**Figure 5:**
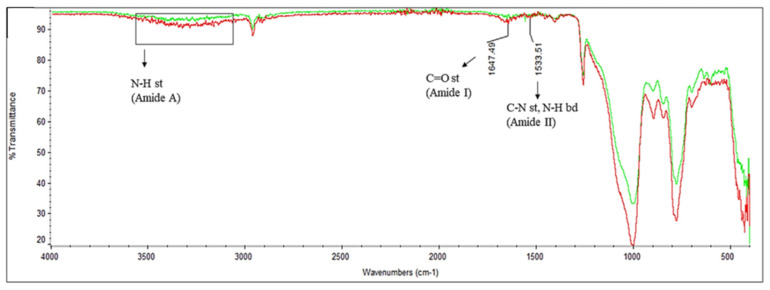
Comparison of ATR/FT-IR spectra of peptide-conjugated catheter surfaces: (**red**) C-PEP, (**green**) PEP-C.

For the immobilization of thiolated peptides onto catheter surfaces, the bands representing the peptide molecules were seen in the FT-IR spectrum as the amide A, amide I, and amide II bending peaks (Figure 5). The amide A peak is located around 3500 cm^−1^ as a broad peak and represents the N-H stretching band. The amide I and II bending peaks are characteristically seen in the double bond region of the FT-IR spectrum, at 1647 and 1533 cm^−1^, respectively.

*Physical characterization*: *Contact Angle Measurement*. The surface hydrophobicity of untreated and C-AMP-treated silicone PDMS surfaces was measured with a contact angle of 0.5 µL dH_2_O. The results of the measurement are given in the table below (Table 2). The CA° of untreated silicone PDMS was measured to be 105.25, indicating that the surface is hydrophobic. After the silicone surfaces are immobilized with the C-AMPs free-radical addition method, their CA° was measured. After immobilizing hydrophilic C-AMPs with the free-radical addition method, the surface CA° was dropped below 90° which means that the surfaces became hydrophilic.

*SEM—Energy dispersion X-ray spectroscopy (EDS) analysis.* Surface images and the semi-quantitative elemental analysis of the C-AMP-immobilized catheters were obtained using SEM-EDS. For EDS analysis, an image of 100 µm area was taken and the elemental compositions were mapped. To compare the elemental compositions of both C-AMPs in free-radical addition (DMPA) methods, the peptide mapping results are given in the table below (Table 3).

**Figure 6:**
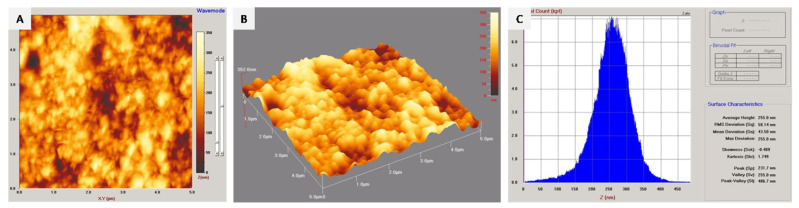
Results of AFM scan for the catheter surface conjugated to C-Pep. (**A**) 2D image, (**B**) 3D image, (**C**) line profile.

*AFM—Atomic Force Microscopy*. The morphology of catheter surfaces conjugated to C-Pep was also analyzed by AFM in order to investigate the uniformity and homogeneity of peptide molecules. In Figure 6, it can be seen that peptide molecules could be immobilized on the catheter surface smoothly and without clumping. Histogram calculation revealed that the average height of the coating layer is around 255 nm.

**Figure 7:**
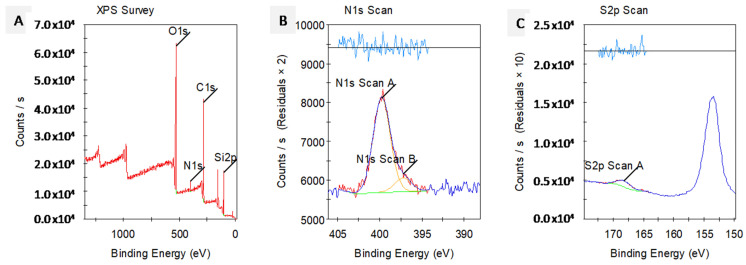
Results of XPS spectra analysis for the catheter surface conjugated to C-Pep. (**A**) XPS survey, high-resolution scans for (**B**) N1s and (**C**) S2p at-oms.

*XPS—X-ray Photoelectron Spectroscopy*. The presence and amount of conjugated peptides on the catheter surface was also investigated by X-ray photoelectron spectroscopy (XPS) analysis. Numerical information is obtained about the atoms and electron states on the surface based on the scattering of photoelectrons by using an X-ray beam that excites solid samples. As seen in Figure 7, the presence of N (nitrogen) atoms as well as C, O and Si atoms is noticeable, and high-resolution scans for N1s and S2p atoms proves the presence of peptide molecules on the silicone surface. Moreover, the ratio for their counts indicates the relative amount of nitrogen and sulfur atoms in the chemical structure of peptide molecules.

### 3.3. Antimicrobial Activity of C-AMP-Immobilized Catheters

**Figure 8:**
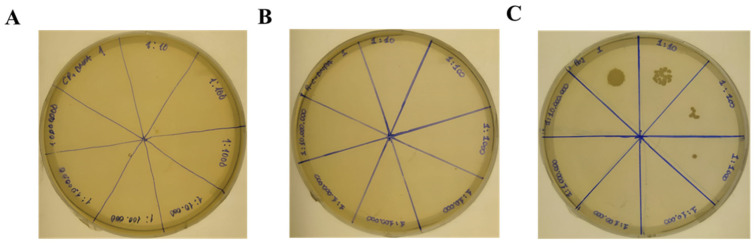
Antimicrobial activity of C-AMPs against ***E. coli 25922***. (**A**) C-Pep; (**B**) Pep-C; (**C**) untreated silicone catheter.

*Colony counting.* For determining the antimicrobial activity, the coated surfaces of the C-AMP-immobilized catheters were submerged into 40 µL of 1 × 10^5^ cfu/mL bacteria inoculum for 40 min. Then, 10 µL of inoculum was serially diluted in the ratio of 1:10 eight times and 10 µL of each dilution was dropped onto an MH agar. After overnight incubation, the visible and countable colonies were counted and the images presented in Figure 8 were evaluated.

When looking at Figure 8, different colony densities at different dilutions can be seen. No colonies were seen in the MH agar plates of C-Pep and Pep-C either (Table 4).

**Figure 9:**
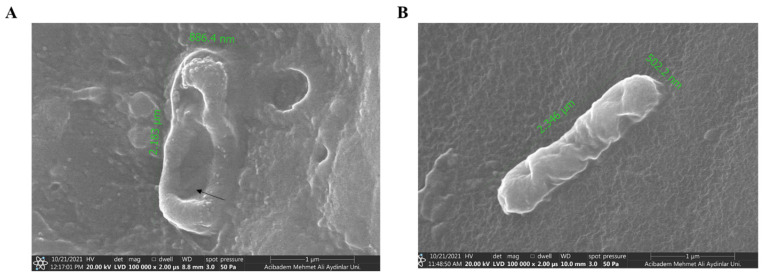
SEM image of *E. coli 25922* on silicone catheter surface under 100,000×. (**A**). Large pore formation of *E. coli* on C-Pep-immobilized surface. (**B**). Normal *E. coli* on untreated catheter surface.

*SEM analysis of E. coli on AMP-immobilized catheter surface*. Pore formation was observed in all bacteria encountered when scanning the surface of the C-Pep-immobilized catheter. Accordingly, it has been observed that the silicone catheter surfaces with immobilized C-Pep antimicrobial peptide gain antimicrobial properties and, in encountering any bacteria, they open holes on the bacterial surface and show a bactericidal effect (Figure 9). The region marked with an arrow indicates the holes observed in the bacterial outer membrane. These images can be interpreted as an indication that the antimicrobial peptides show activity even after binding to the catheter surface, and that bacteria trying to attach to the surface succeed in opening holes in the outer membranes and successfully provide bacterial death.

## 4. Discussion

AMPs can be naturally found in the immune system of living organisms, or they can be designed synthetically [41]. The AMPs that are used in this work are designed based on the amphipathic nature of the natural AMPs. The sequence of thiolated AMP consists of 17 amino acids, in which 6 AA residues are positively charged R in L-form, 10 amino acid residues are hydrophobic L in D-form, and 1 amino acid residue is C in L-form located at the N- or C-end of the peptide sequence. The C-AMPs were synthesized with solid-phase peptide synthesis. In both sequences (C-PEP and PEP-C), the ending was an amide group which contributes overall to the net positive charge of the peptides. After the synthesis process, the C-AMPs were analyzed with LC-MS/MS instrument for their chemical structure and molecular weight. The C-AMPs have a net +7 charge insixof them due to the six R residues in the peptide sequence and the remaining +1 charge is due to the amide end. When looking at the positive scan of LC-MS/MS spectrum for C-PEP, five out of seven expected fragments *m*/*z* values are 365, 438, 730, and 1095 (gmol^−1^) corresponding to +2, +3, +4, and +5 charges can be detected. For the PEP-C, in positive scan, only a +2 charged fragment that has *m*/*z* value 365 gmol^−1^ was seen in the spectrum.

The purity of the synthesized peptides was evaluated with rt-HPLC. The two peaks can be either due to the possible disulfide bond in between C-AMPs dimer or due to the different protonation degrees caused by TFA in the mobile phase. To ensure that there was no disulfide bond formation after the cleavage of the peptides, before and after DTT treatment; Ellman’s assay was conducted to measure the free sulfhydryl group amount. The results indicate there is no disulfide bond between C-AMPs, which means that the initially detected two peaks in both C-PEP and PEP-C HPLC chromatograms were due to the different amounts of protonated versions for the same peptide molecule.

The antibacterial activity of the C-AMPs was determined with MIC, where the thiolated versions of the PEP have higher MIC values yet are still lower than ampicillin which was used as a positive control. Compared to C-PEP and PEP-C together, C-PEP was found to be more effective against both gram-positive bacteria *S. aureus* strains and gram-negative *E. coli* strains. The cytotoxic effect of the C-AMPs was analyzed with MTT assay. In all three cell types, non-thiolated PEP showed more toxic effects than the C-AMPs. When comparing the MIC values of C-PEP, which are 1 µg/mL and 2 µg/mL with the MTT results, it can be said that the effective concentrations against bacteria were found be non-toxic for the 3T3 and HeLa cell lines. On the other hand, for the HaCat cell line, 2 µg/mL concentrations were found to be in the 50% toxicity range. For PEP-C, the MIC value was 8 µg/mL, which was shown be to lower than the 50% toxicity range in all three cell lines. Similar to the MTT test results, the effective MIC values did not show a hemolytic effect against freshly collected human red blood cells.

The characteristic chemical groups of peptide molecules (C=O, and N-H of amide) were detected for C-AMP-immobilized catheter surfaces with ATR/FT-IR spectroscopy. The three major bonds that are determined in the C-AMP-coated silicone catheter surfaces are amide A, amide I, and amide II. Comparing the amide A, amide I, and amide II spectral bands with each other, it can be concluded that the more peptide molecules that were immobilized onto the catheter surface, the larger the spectral bands become [42]. The difference in the amide I stretching band is particularly noticeable, indicating that more peptides were immobilized on the catheter surface using the PEP-C peptide.

For the surface hydrophobicity determination, contact angle (CA°) measurements were applied to the peptide-immobilized catheter pieces in the dimensions of 0.5 cm × 0.5 cm against a 0.5 µL drop of dH_2_O that was used each time. While an untreated PDMS surface gives a contact angle of 105, after the peptide immobilization the expected CA° measurement was a decrement in the water angle even though the peptides were observed at around 60% hydrophobicity in HPLC analysis. Although peptides could be counted as hydrophobic, they are still more hydrophilic than untreated PDMS surfaces, so the CA° of C-AMP-immobilized PDMS surfaces should be lower than 100° and are predicted to become more hydrophilic. The difference in the angles could be due to surface irregularities. Since PDMS molds were prepared by hand, they were not perfectly flat, which might affect the CA° measurement. Still, the obtained results were coherent with the ATR/FT-IR spectra, so it can be interpreted that more peptides can be immobilized to hydrophobic silicone surfaces via UV-assisted thiol-ene reaction.

Semi-quantitative elemental analysis of the peptide-immobilized silicone catheter surfaces was performed with EDS detector-assisted SEM. The expected types of elements in the EDS mapping are carbon (C), oxygen (O), nitrogen (N), and especially sulfur (S) due to the sulfhydryl group at C amino acid residue. The reason for specifically searching for S atom and its amount is significant since its presence indicates successful C-AMP-immobilization onto silicone catheter surfaces. The atomic percentage of elements was compared and the results were as anticipated; S atoms were detected almost two times more for the samples obtained via free-radical addition reaction compared to those modified by nucleophilic addition reaction.

Visualization of catheter surfaces bound to C-PEP was also evaluated by AFM microscopy, which demonstrates that the coating thickness on surfaces was around 255 nm. The height and coating thickness did not change significantly across the analyzed surface area and the peptide molecules are homogeneously bound to the surface. Also, XPS analysis for the catheter surface conjugated to C-PEP revealed that there are Nitrogen atoms (N1s peak) on the surface, accounting for3.78%, together with O1s (28.96%), C1s (47.76%) and Si2p (19.54%), which verifies the presence of peptide molecules. Together with the peak seen at a binding energy of 400 eV for N1s, there are also sulfur atoms detected as S2p, appearing at 167 eV. These spectra clearly prove that the peptide molecules have been attached to the catheter surface via their thiol groups during the radicallic thiol-ene click reaction.

After verifying all four atoms, C, O, N, and S, the antimicrobial activity of the peptide-immobilized catheter surfaces was investigated by treating the coated surface with a certain colony of bacteria for colony counting. Coated surfaces were treated with 1 × 10^5^ cfu/mL *E. coli*, *ATCC 25922*, for 40 min at 37 °C. After bacteria treatment, the inoculum was serially diluted at the ratio of 1:10 and 10 µL of this solution was dropped onto an MH agar plate. After overnight incubation, it was seen that for the untreated catheter sample, bacteria continue to grow and at 10^−2^ dilution, seven colonies can be counted, which was expected compared to the untreated control surface. For the MH agar plate belonging to the untreated catheter pieces, it was seen that there was not any countable colony formation for C-PEP and PEP-C. This was an expected result since all the surface characterization analysis free-radical addition immobilization was found to be an effective method. With radicallic thiol-ene reaction, the high number of peptides can be immobilized to the catheter surface and peptides can preserve their anti-bactericidal effect.

## 5. Conclusions

Novel antimicrobial peptides (AMPs) with thiol functionalities were prepared via SPPS with a high purity of over 90% and utilized for the elimination of hospital-associated infections originated from silicone catheter usage. Peptides were modified with a cystein aminoacid from either end (C-PEP for N terminus and PEP-C for C terminus), so that they could be conjugated to the catheter surface in two different orientations, separately. The cytotoxicity of these modified AMPs was investigated against mammalian cells, (3T3, HaCat, and HeLa), showing non-toxic behavior at their MIC values. The surface modification of silicone catheters was successfully achieved by the generation of hydroxyl groups upon UV/ozone treatment, and silane molecules with allyl groups were attached afterward. Radicallic thiol-ene chemistry, which took place between the thiol groups of AMPs and the allyl functionalities on the catheter surface, resulted in a peptide-immobilized surface. While FT-IR spectroscopy and XPS analysis chemically prove the presence of peptides on the catheter surface, the contact angle decreases from 105.25 to 65.10 for C-PEP and 73.89 for PEP-C, and AFM images demonstrate the physical changes on the surface upon peptide conjugation. Compared to the untreated original catheter, an antibiogram test performed against *E. coli 25922* revealed the more effective antimicrobial property of peptide-immobilized catheter pieces forming no colonies on the surface. Moreover, the distorted morphological changes in *E. coli 25922* on C-PEP modified catheter surface, compared to the untreated one, clearly indicate the successful and beneficial immobilization of these novel AMPs on the catheter surface. These findings pave the way for the application of this thiol-ene-based conjugation strategy to attach biomolecules to the surface of silicone-based medical devices, such as different type of catheters and silicone drainage systems, for the elimination of further possible infections, directly referring to public health.

## Figures and Tables

**Table 1:** MICs of C-Pep and Pep-C against different bacteria strains.

Bacterial Strain	C-Pep (µg/mL)	Pep-C (µg/mL)	Pep (µg/mL)
*E. coli (NCTC 13846)*	1	8	0.5
*E. coli (ATCC 25922)*	2	8	0.5
*S. aureus (ATCC 29213)*	1	8	0.5
*S. aureus (ATCC 25923)*	2	8	0.5
*MRSA*	2	8	0.5

**Table 2:** Contact angle measurements of distilled water on C-AMP-treated and untreated silicone PDMS surfaces.

Sample	Contact Angle (CA°)
PDMS	105.25
C-Pep	65.10
Pep-C	73.89

**Table 3:** Atomic percentage of elements obtained from EDS analysis for peptide immobilization.

Element	C-Pep	Pep-C
C	14.93	15.02
N	2.70	3.13
O	80.39	80.26
S	1.98	1.59

**Table 4:** Colony counts of C-AMP-treated and untreated catheter surfaces against *E. coli*.

	Colony Count (cfu)		
Initial Bacterial Concentration (cfu/mL)	Untreated Catheter	C-Pep Immobilized Catheter	Pep-C Immobilized Catheter
1 × 10^5^	7	0	0

## Data Availability

The data used to support the findings of this study will be available from the corresponding author upon request.

## Supplementary Materials

The following supporting information can be downloaded at: https://www.mdpi.com/article/10.3390/pharmaceutics16081045/s1, Figure S1: C-P1 RP-HPLC chromatograms with scanning method. A. C-P1 without DTT treatment. B. C-P1 0.1% (*w*/*v*) DTT treatment; Figure S2: P1-C RP-HPLC chromatograms with scanning method. A. P1-C without DTT treatment. B. P1-C 0.1% (*w*/*v*) DTT treatment; Figure S3: ATR-FT-IR spectrum of the plain silicone catheter surface (st = stretching); Figure S4: ATR/FT-IR spectrum of 2 h UV/Ozon and 30 min 2 mM GSH treated silicone catheter (st = stretching); Table S1: Net charge and corresponding *m*/*z* values for C-AMPs.
