# Supplementary material for: Effective Immobilization of Novel Antimicrobial Peptides via Conjugation onto Activated Silicon Catheter Surfaces
Source: Pharmaceutics. 2024 Aug 6;16(8):1045. doi: 10.3390/pharmaceutics16081045 (PMC11360073; doi:10.3390/pharmaceutics16081045)
Supplement: Supplementary file 1 [file pharmaceutics-16-01045-s001.zip › pharmaceutics-3136076-SI.pdf]

Article

Supplementary Materials: Effective Immobilization of Novel Antimicrobial Peptides via Conjugation onto Activated Silicon Catheter Surfaces

Irem Soyhan, Tuba Polat, Erkan Mozioglu, Tuğba Arzu Ozal Ildenız, Merve Acikel Elmas, Sinan Cebeci, Nihan Unubol, and Ozgul Gok

1. RP-HPLC results of DTT treated C-AMPs

The control of disulfide bond (S-S) formation between the sulfhydryl group of C-AMPs, peptides were treated with 0.1 % (w/v) DTT for 1.5 hours and analyzed with the same scanning method.

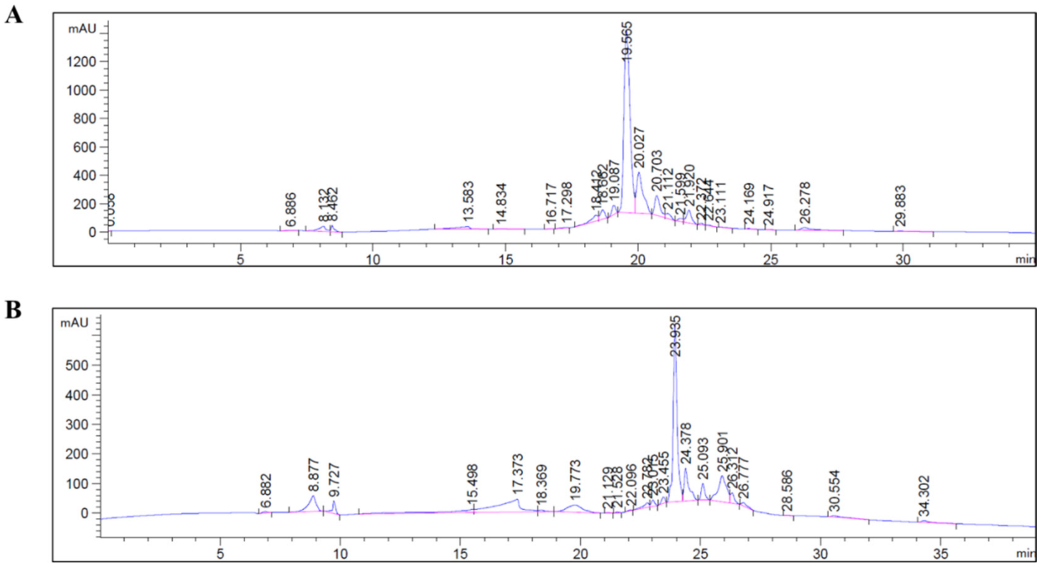

Figure S1. C-P1 RP-HPLC chromatograms with scanning method. **A.** C-P1 without DTT treatment. **B.** C-P1 0.1% (w/v) DTT treatment.

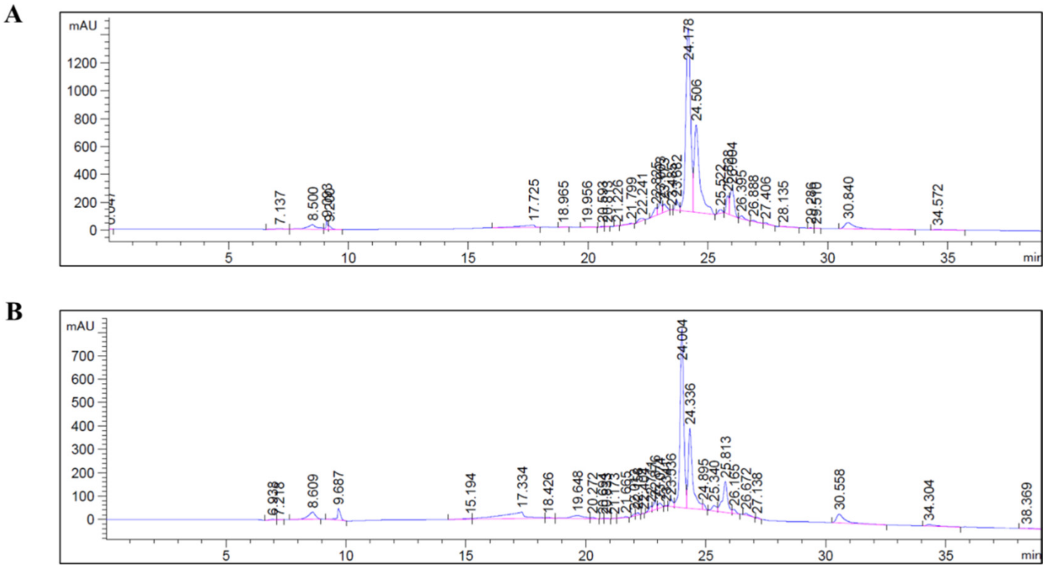

Figure S2. P1-C RP-HPLC chromatograms with scanning method. **A.** P1-C without DTT treatment. **B.** P1-C 0.1% (w/v) DTT treatment.

2. Mass/Charge values of C-AMPs For LC-MS/MS Analysis

Table S1. Net charge and corresponding m/z values for C-AMPs

Mass (m)	Charge (z)	Mass/Charge (m/z)
	+1	2189.89
	+2	1095.45
	+3	730.63
2188.89	+4	547.97
	+5	438.58
	+6	365.82
	+7	313.70

3. FT-IR Analysis of Silicone Catheter Pieces

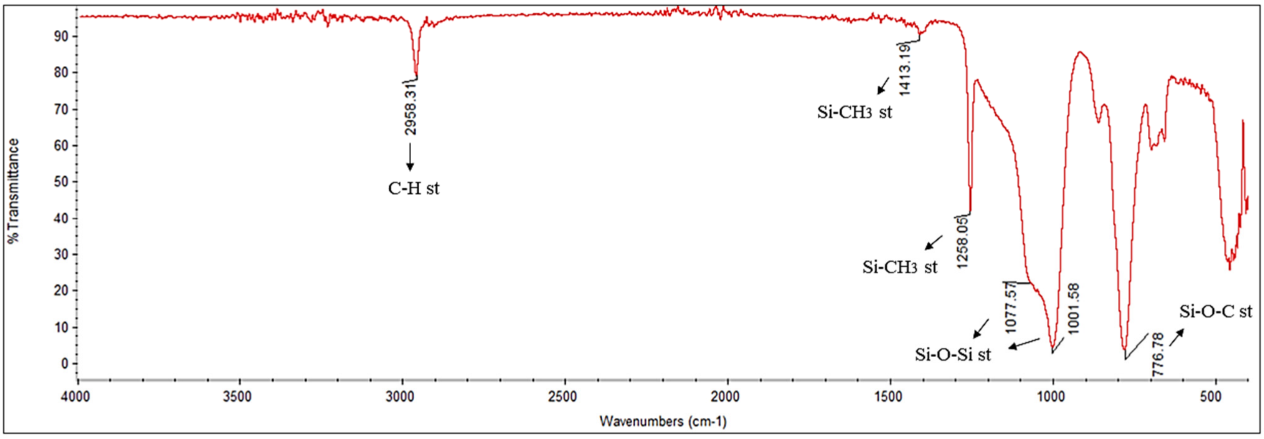

Figure S3. ATR-FT-IR spectrum of the plain silicone catheter surface (st=stretching)

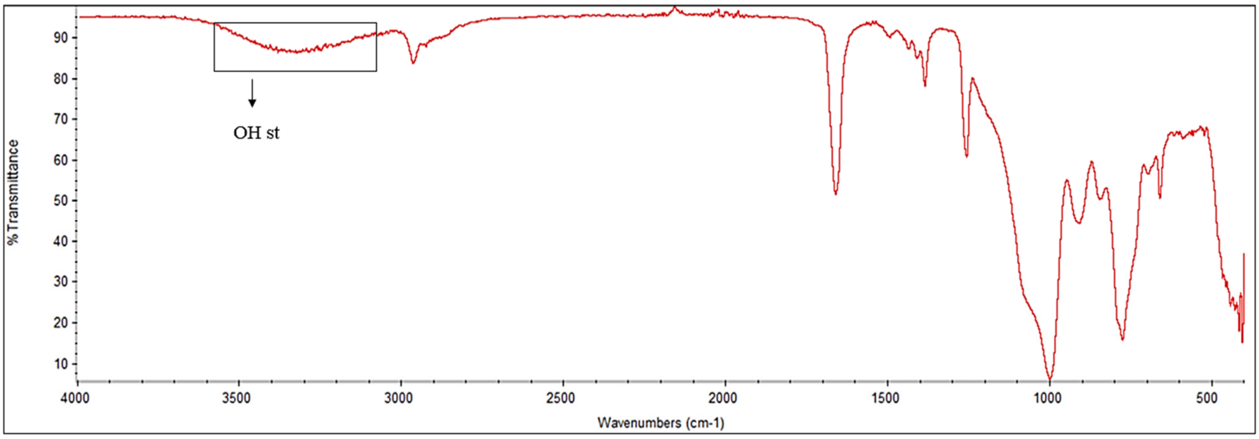

Figure S4. ATR/FT-IR spectrum of 2 hours UV/Ozon and 30 minutes 2 mM GSH treated silicone catheter (st=stretching).
